# Fermentation of Nocellara Etnea Table Olives by Functional Starter Cultures at Different Low Salt Concentrations

**DOI:** 10.3389/fmicb.2018.01125

**Published:** 2018-06-05

**Authors:** Alessandra Pino, Maria De Angelis, Aldo Todaro, Koenraad Van Hoorde, Cinzia L. Randazzo, Cinzia Caggia

**Affiliations:** ^1^Department of Agricultural, Food and Environment, University of Catania, Catania, Italy; ^2^Department of Soil, Plant and Food Sciences, University of Bari Aldo Moro, Bari, Italy; ^3^Department of Agricultural, Food and Forest Science, University of Palermo, Palermo, Italy; ^4^Laboratory of Brewing and Biochemistry, Faculty of Bioscience Engineering, Ghent University, Ghent, Belgium

**Keywords:** NaCl content, probiotic strain, metabolomics, microbiota, REP-PCR analysis

## Abstract

Nocellara Etnea is one of the main Sicilian cultivars traditionally used to produce both olive oil and naturally fermented table olives. In the present study, the effect of different salt concentrations on physico-chemical, microbiological, sensorial, and volatile organic compounds (VOCs) formation was evaluated in order to obtain functional Nocellara Etnea table olives. The experimental design consisted of 8 treatments as follow: fermentations at 4, 5, 6, and 8% of salt with (E1-E4 samples) and without (C1-C4 samples) the addition of starters. All the trials were carried out at room temperature (18 ± 2°C) and monitored for an overall period of 120 d. In addition, the persistence of the potential probiotic *Lactobacillus paracasei* N24 at the end of the process was investigated. Microbiological data revealed the dominance of lactic acid bacteria (LAB), starting from the 7th d of fermentation, and the reduction of yeasts and enterobacteria in the final product inoculated with starters. VOCs profile highlighted a high amount of aldehydes at the beginning of fermentation, which significantly decreased through the process and a concomitant increase of alcohols, acids, esters, and phenols. In particular, esters showed an occurrence percentage higher in experimental samples rather than in control ones, contributing to more pleasant flavors. Moreover, acetic acid, ethanol, and phenols, which often generate off-flavors, were negatively correlated with mesophilic bacteria and LAB. It is interesting to note that salt content did not affect the performances of starter cultures and slightly influenced the metabolome of table olives. Sensory data demonstrated significant differences among samples registering the highest overall acceptability in the experimental sample at 5% of NaCl. The persistence of the *L. paracasei* N24 strain in experimental samples, at the end of the process, revealed its promising perspectives as starter culture for the production of functional table olives with reduced salt content.

## Introduction

The greater consumer's attention for healthy food is confirmed by the growing trend in fermented vegetables consumption, such as table olives (International Olive Council (IOC), 2016). Table olives are mainly produced in several Mediterranean countries, such as Spain, Italy, and Greece and in Sicily, two main cultivars (Nocellara del Belice and Nocellara Etnea) are growth. In particular, Nocellara Etnea cv is mainly cultivated in the Central and Eastern area of Sicily, among the provinces of Enna, Catania, Messina, Syracuse, and Ragusa. The drupes, elliptical in shape and slightly asymmetric, are characterized by a uniform and a large size and by late harvesting. The relationship between core and pulp is very high and this character makes this cultivar one of the best for the production of green table olives. The latter are mostly obtained by a spontaneous process in which the hydrolysis of oleuropein is relied on enzymatic activities of indigenous microorganisms, and on the plasmolytic effect of salt. This process is mainly dominated by lactic acid bacteria (LAB) and yeasts, which form a natural consortium (Randazzo et al., 2010). However, during the spontaneous fermentation spoilage microorganisms, such as *Enterobacteriaceae* and *Propionibacteriaceae* may occur. It is well established that *Lactobacillus plantarum* and *Lactobacillus pentosus* are the main detected species, due to their versatile adaptation to the brine environment (Ruiz-Barba et al., 1994; G-Alegria et al., 2004; Bautista-Gallego et al., 2010; Randazzo et al., 2011, 2012; Hurtado et al., 2012; Cocolin et al., 2013; Tofalo et al., 2014), and different strains are widely used as starter cultures in several table olive fermentations (Arroyo-López et al., 2012; Hurtado et al., 2012; Randazzo et al., 2014).

During olive fermentation, coarse salt is added in order to reduce the water activity, preventing the growth of spoilage microorganisms, and to improve taste and textures of the final product (Bautista-Gallego et al., 2013a). The EU Member States try to implement national nutritional policies with the aim to decrease salt intake according to the European Commission suggestion (European Council, 2010). The strategy to set up table olives with a reduced daily Na intake, which has been established at 5 g salt, by WHO/FAO [World Health Organisation (WHO)/Food 2003] is one of the main goals of food industry. Different chloride salts, such as KCl, CaCl_2_, and ZnCl_2_, have been evaluated as replacers for NaCl (Bautista-Gallego et al., 2013b), especially in Spanish style green olives (Bautista-Gallego et al., 2010, 2011). The reduction in Na and the increase in other salts may lead to a more equilibrated mineral composition in table olives, ameliorating the consumers' diet, and enhancing the perception of the nutritional value of the olives. Nevertheless, the effects of NaCl replacement with other salts could affect the microbiota evolving in the fermentation process of table olives (Bautista-Gallego et al., 2015; Mateus et al., 2016), as well as impact the sensorial quality of the final product (Zinno et al., 2017). Potential NaCl reduction depends on characteristics linked with the cultivar, its composition, other ingredients, processing, and technological parameters (Bautista-Gallego et al., 2013a), which should be well addressed before their implementation at the industrial scale. Furthermore, the final product must be safe from the microbiological point of view. It is already established that a reduction in NaCl might be responsible for an increase of pathogens such as *Clostridium botulinum* (Simpson et al., 1995).

Nowadays, based on the increasing consumers' demand, the production of healthier table olives is of great industry importance, taking into account the potential market of table olives as a functional food. In fact, functional table olives can provide a concrete opportunity to convey the benefits that are already appreciated by consumers in dairy sectors. It has already been demonstrated that table olives represent a good vehicle to transport probiotics to humans for both their microarchitecture and the presence of nutrients (Lavermicocca et al., 2005; Valerio et al., 2006; Randazzo et al., 2017).

The aim of the present study was (i) to set up a fermentation, at laboratory scale, of Nocellara Etnea table olives with reduced level of NaCl; (ii) to evaluate the effect of the NaCl reduction on the physico-chemical, microbiological, and sensorial parameters compared to fermentation carried out without starter cultures.

## Materials and methods

### Bacterial strains and olive processing

In the present study two lyophilized LAB strains, *L. plantarum* UT2.1 and the potential probiotic *Lactobacillus paracasei* N24, belonging to the Di3A microbial collection, previously screened for their technological and functional features, and already applied as starter cultures at industrial scale (Randazzo et al., 2017) were used. Each strain was directly inoculated into fresh brine (1:1 ratio) to reach a final cell density of 7 log colony forming units per ml (cfu/ml). Olives of Nocellara Etnea cultivar, kindly provided by a local company (Consoli srl, Adrano, Sicily) were processed, at laboratory scale, following the Sicilian style method. After harvesting, about 3 kg of olives were subjected to quality control, to remove damaged fruits, washed with tap water, directly placed in sterile glass vessels, and covered with ~3 l of sterile brine. The experimental design consisted of 8 treatments as follows: fermentations at 4, 5, 6, and 8% of salt with the addition of starters (E1-E4 samples); fermentations at 4, 5, 6, and 8% of salt without the addition of starters (C1-C4 samples). All fermentation trials were carried out at room temperature (18 ± 2°C), and monitored for an overall period of 120 d. The brine salt concentration was maintained at each initial level by adding marine salt. Fresh brine was periodically supplied to maintain olives totally dipped in order to inhibit growth of molds on the brine surfaces. The experimental trials were carried out in triplicate. The progression of the fermentation was followed by monitoring pH, titratable acidity and the shift in microbial populations in brine throughout the process.

### Physico-chemical and total polyphenol determination of brine samples

Fifty ml of each brine sample were taken at 60 and 120 days of fermentation. The pH values of brines were monitored by a pH meter (H19017, Microprocessor, Hanna Instruments). Total free acidity was measured by titration and expressed as the percentage of lactic acid (g/100 ml brine). Total polyphenol content was colorimetrically determined in brine samples at 60 and 120 d, using Folin-Ciocalteu reagents, according to Singleton (1974). Polyphenols were measured in triplicate and expressed as mg/l of gallic acid.

### Microbiological analyses of brine samples

For the microbiological characterization, brine samples were analyzed at 1, 7, 15, 30, 60, 90, and 120 days of fermentation. At each sampling time, brines were serially diluted, using sterile quarter-strength Ringer's solution (QRS), and plated in triplicate on the following agar media (all provided from Oxoid Italy), and conditions: Plate Count Agar, incubated at 32 ± 2°C for 48 h, for total mesophilic bacteria; de Man-Rogosa-Sharp agar, supplemented with cycloheximide (5 ml/l), anaerobically incubated at 32°C for 24–48 h, for LAB count; Sabouraud Dextrose Agar, supplemented with chloramphenicol (0.05 g/l), incubated at 25°C for 4 days, for yeast count; Violet Red Bile Glucose Agar, aerobically incubated at 37°C for 24 h, for Enterobacteriaceae count; Mannitol Salt Agar, incubated at 32°C for 48 h, for staphylococci enumeration; Mac Conkey incubated at 32°C for 24–48 h for *Escherichia coli* determination. Results were expressed as log_10_ cfu/ml.

### Volatile organic compound (VOC) analysis by gas chromatography-mass-spectrometry (GC-MS)

VOCs, detected in brine samples at 1, 60, and 120 d of fermentation, were sampled using a solid-phase microextraction (SPME). The SUPELCO SPME (Bellefonte, PA) fiber holder and fiber used were coated with divinylbenzene/polydimethylsiloxane (DV/PDMS), 65 mm. Before the first extraction, the fiber was conditioned in the GC injector port at 300°C for 1 h, according to the manufacturer's recommendation. Ten ml of brine sample were added to a 35 ml vial. Extraction temperature of head-space and time were 40°C and 20 min, respectively. One g of NaCl was added to increase extraction rate of VOCs. The samples were gently vortexed during extraction using a magnetic stirrer. Fiber exposition was prolonged for 20 min at 40°C. Thermal desorption was performed in the injector at 230°C for 1 min (Sabatini et al., 2008; Malheiro et al., 2011). The identification of the extracted VOCs was carried out using a GC instrument (HP GC6890, Hewlett Packard, Palo Alto, CA), coupled to a MS detector (HP MS5973) (Panagou and Tassou, 2006). The gas chromatograph was equipped with a 30 m 0.25 mm i.d. 0.25 mm film thickness fused-silica capillary column (DB-WAX J&W Scientific) and the injector temperature was 230°C. The conditions applied were as those previously reported (Randazzo et al., 2017). The quantification of VOCs was determined with the internal standard method spiking propionic acid, ethanol, ethyl acetate, benzaldehyde and guaiacol to all analyzed samples. All analyses were performed in duplicate and the results were expressed in μg/l of brine.

### Isolation and genetic identification of lactic acid bacteria

From each MRS agar plate, at the highest dilution, of both E (E1-E4) and C (C1-C4) brine samples at 1, 60, and 120 d of fermentation, the 20% of total number of colonies was randomly selected, purified, checked for catalase activity and Gram reaction, and microscopically examined before storing in liquid culture using 20% glycerol at −80°C. The random colony selection from the highest dilution plates allowed us to collect 400 LAB isolates. Total genomic DNA of isolates was extracted from overnight cultures according to the method described by Pino et al. (2018). DNA concentration and DNA quality were assessed by measuring optical density using Fluorometer Qubit (Invitrogen, Carlsbad, 278 CA, USA).

All LAB isolates were subjected to *Rec*A and *Tuf* gene species-specific PCR following the protocol previously described by (Torriani et al., 2001; Ventura et al., 2003).

### REP-PCR analysis

In order to evaluate the viability of the potential probiotic *L. paracasei* N24 throughout the fermentation, lactobacilli isolated from E samples at 60 and 120 d (60 isolates), ascribed to *L. paracasei* species through the aforementioned species-specific multiplex PCR, were subjected to REP-PCR analysis, using the (GTG)_5_-primer, as described by Gevers et al. (2001). PCR amplicons were separated on a 1.5% agarose gel (w/v) in 1X TAE buffer (40 mM Tris, 20 mM acetic acid and 1 mM EDTA) under highly standardized conditions (55 V, 400 mA, 16 h at 4°C). At regular intervals a reference marker (6 μl each composed of 1.10 μl Molecular Ruler 500 bp (Bio-Rad), 1.40 μl Molecular Ruler 100 bp (Bio-Rad), 2 μl TE buffer (1 mM EDTA, 10 mM Tris–HCl, pH 8.0) and 1.50 μl loading dye), was loaded for normalization. Profiles were visualized under ultraviolet light, after staining with ethidium bromide. Digitized images of gels were normalized and analyzed by the BioNumerics 7.6.2 software (Applied Maths, Belgium). Similarity matrices of densitometric curves of the gel tracks were calculated with Pearson's product-moment correlation coefficient. Subsequent cluster analyses of similarity matrices were performed by unweighted pair group method with arithmetic averages (UPGMA).

### Table olives sensory evaluation

The sensory assessment of table olives was performed by a trained sensory panel consisting of 10 panelists (6 females and 4 males, aged from 22 to 40 years), according to the method reported by the International Olive Council (International Olive Council (IOC), 2011). Olives were tasted for negative sensations (abnormal fermentation such as musty, rancid, cooking effect, soapy, metallic, earthy, and winey-vinegary), based on the classification reported by International Olive Council (IOC) (2011, 2016), while a descriptive analysis was carried out for descriptors corresponding to gustatory sensations (acidity, saltiness, and bitterness) and kinaesthetic sensations (hardness, fibrousness, and crunchiness). In addition, an overall acceptability descriptor was considered as an indication of the overall quality. Sensory data were acquired by a direct computerized registration system (FIZZ Biosystemes. Couternon, France).

### Statistical analysis

Statistical analysis of chemical data was performed using a one-way analysis of variance with repeated measures of the GLM procedure by SAS (2001), considering the different treatments as variable. Means were separated by a Least Significant Difference (LSD) test when a significant treatment (*P* < 0.05) was observed. Microbiological data and VOCs were analyzed by ANOVA (One-way Analysis of Variance) using Tukey's *post-hoc* test, in order to assess the overall differences among samples. Statistical analysis was performed using XLSTAT PRO 5.7 (Addinsoft, New York, USA) and the reference level of significance was 0.05 in all the assays. Sensory data were submitted to one-way ANOVA using the software package Statgraphics® Centurion XVI (Statpoint Technologies, INC.) using samples as treatments. The significance was tested by means of the F-test. To differentiate the samples, the mean values were submitted to the multiple comparison test using the LSD procedure. In order to correlate the experimental and control brine samples to volatile compounds, data obtained at 1, 60, and 120 d of fermentation were subjected to principal component analysis (PCA) using MATLAB, achieving high data compression efficiency of the original data.

Similarities between the microbiota and metabolome profiles of experimental and control brine samples were carried out by PermutMatrix software. Data correlations between microbiota (mesophilic bacteria, LAB, staphylococci, yeasts, enterobacteria) and VOCs were computed using Statistica v. 7.0 and elaborated through PermutMatrix software.

## Results

### Physico-chemical data

**Figure 1:**
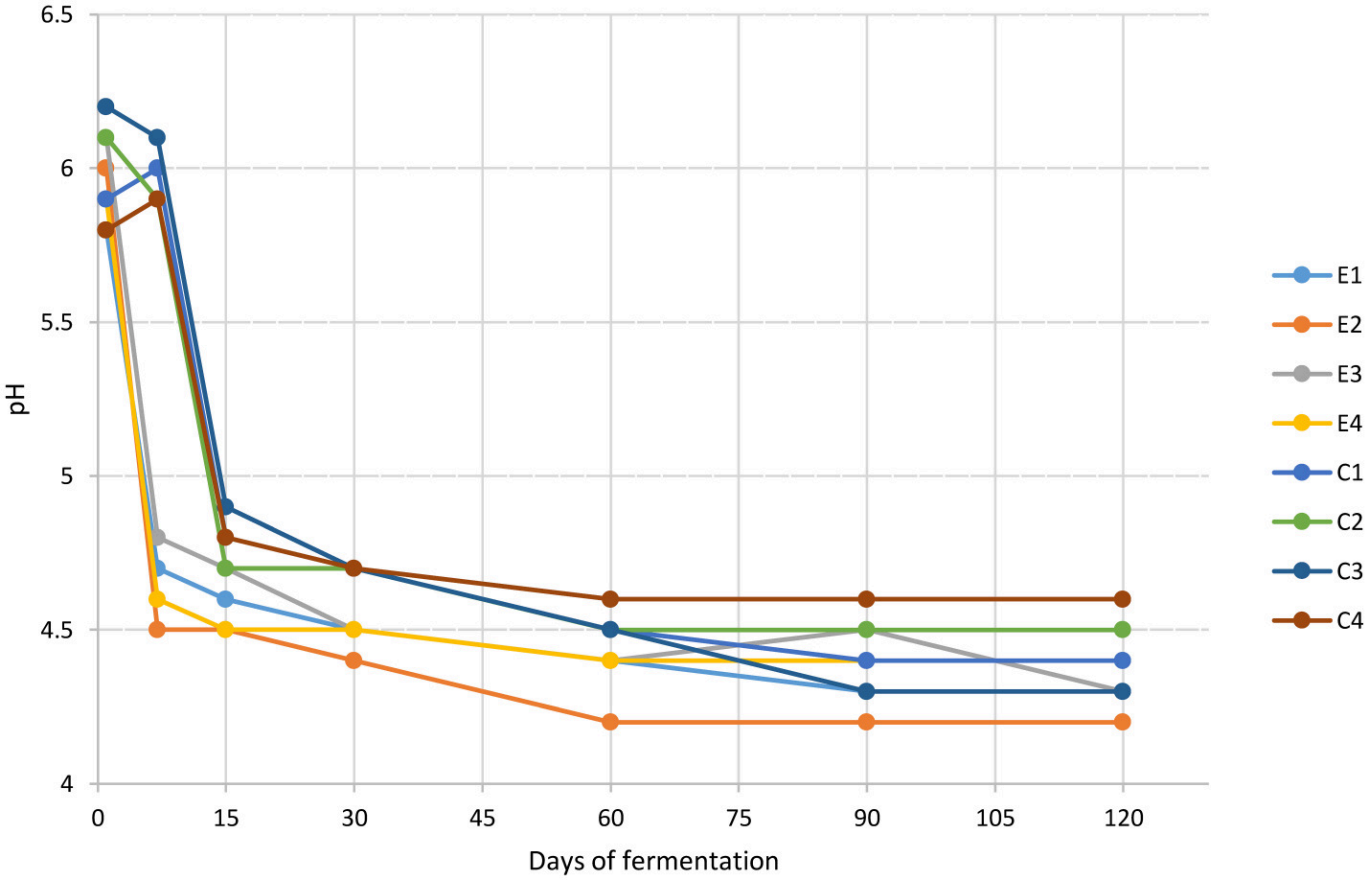
Changes in pH throughout the fermentation of Nocellara Etnea experimental and control samples.

**Figure 2:**
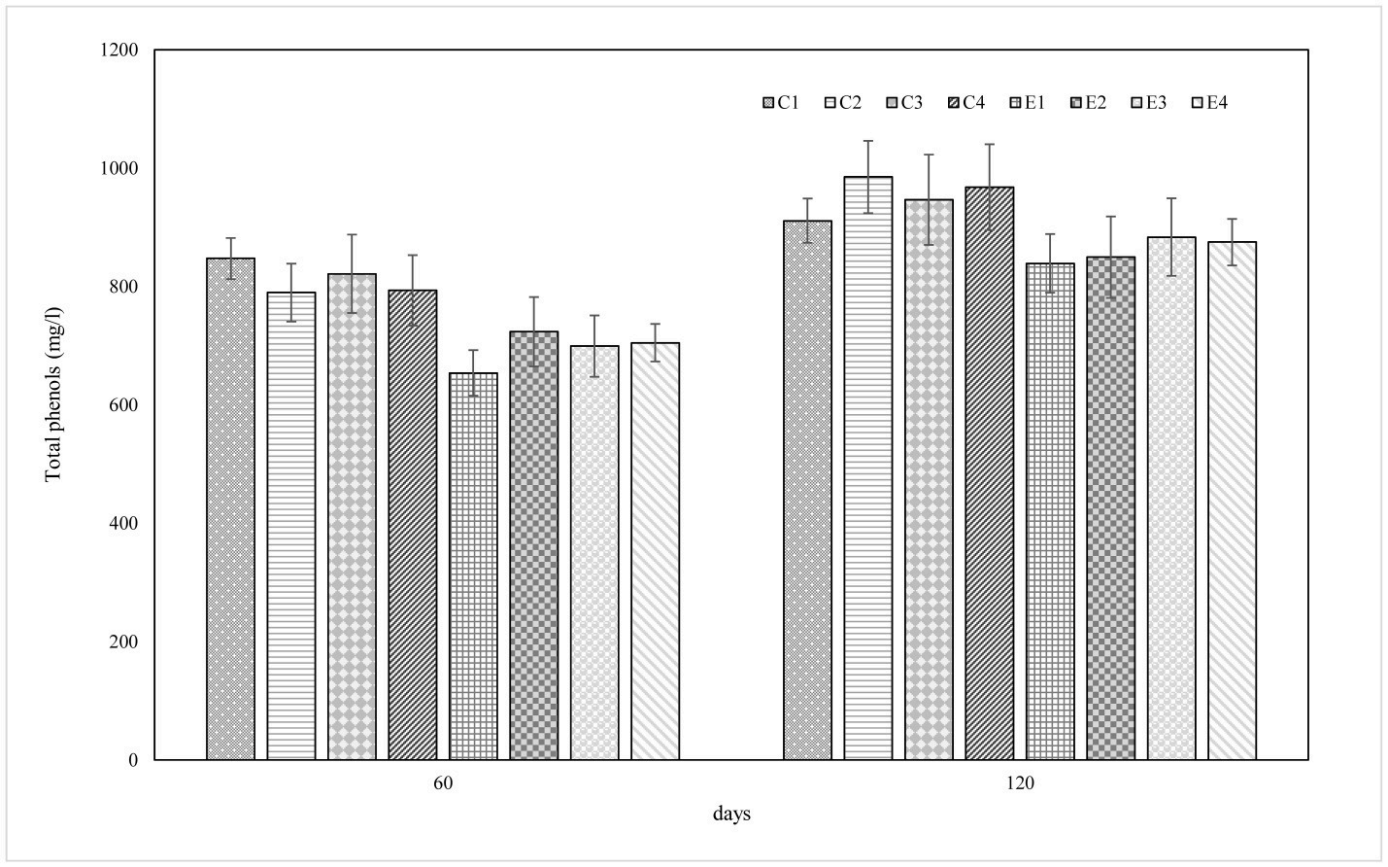
Total phenolic content (mg/l) of control (C) experimental (E) brine samples at 60 and 120 days of fermentation.

The pH values, showed in Figure 1, dropped faster in experimental samples rather than in control ones reaching a value between 4.5 and 4.8 at 7 days of fermentation. The lowest pH value (4.2) was revealed by E2 sample at 60 days. At the end of the process, pH values ranged from 4.6 to 4.2. The titratable acidity, expressed as the percentage of lactic acid (g/100 ml brine), was determined at 60 and 120 d of fermentation. Overall, no differences were detected among experimental and control samples and results showed an increase during the fermentation process, reaching an average value of 0.37 g/100 ml brine in the final products (data not shown). Results of total phenols (TP) content are presented in Figure 2. TP detected in brines showed quite differences among E and C samples while within each group similarities on TP content was achieved. In addition, statistically significant differences were noticed between samples at 60 and 120 d of fermentation with a range of 911.2–985.2 mg/l for C samples and of 839.2–967.8 mg/l for E samples.

### Microbial counts of brine samples through the fermentation

Microbial counts, expressed as log_10_ cfu/ml, of both experimental (E) and control (C) brine samples, at different salt concentrations (4, 5, 6, and 8%), are reported in Table 1. Viable mesophilic bacteria exhibited different behavior among samples. In detail, a steady trend in samples inoculated with starter cultures (E1-E4), was recorded, with the exception of E4 sample (at 8% of NaCl), in which, from a lower initial value (6.65 log unit), a slight decrease throughout the fermentation was observed. Control samples (C1-C4) exhibited a mean initial value of 4.6 ± 0.07 log unit, which significantly increased from the 15th day of fermentation, reaching a final mean value of 5.43 ± 0.09 log unit (Table 1). Similar behavior was observed for LAB population, which reached the highest concentration value in all inoculated samples (E1-E4). The highest value (8.58 log unit) was detected after 60 days of fermentation in sample E2, obtained adding 5% of salt. Among the control samples (C1-C4), a similar initial LAB count was found (average value of 4.4 ± 0.20 log cfu/ml) with the exception of C4 sample, which presented the lowest LAB cell density (Table 1). At the end of the fermentation (120 days) no statistically significant differences (*P* > 0.05) were achieved among samples, that exhibited an average value of 5.88 ± 0.15 log cfu/ml. Yeasts were present at an initial average level of 3.93 ± 0.08 log cfu/ml and 4.18 ± 0.17 log cfu/ml, in E and C samples, respectively. These densities slightly increased through the fermentation process, achieving, at 120 days, an average value of 5.51 ± 0.13 log unit, with the exception of samples E1, E2, and E3, which exhibited the lowest yeast count (Table 1). At the beginning of fermentation, the staphylococci level was quite similar among inoculate and un-inoculated samples, with a slight increase at 30 and 60 days, and followed by a decrease to a final average value of 3.53 ± 0.19 cfu/ml. Enterobacteria counts significantly decreased from the 30th day of fermentation, reaching a final value below 2 log. In addition, *E. coli* was never detected in any brine samples analyzed.

**Table 1:** Microbial counts expressed as log_10_ CFU/ml of 3 replicates ± standard deviation of the main microbial groups detected in Nocellara Etnea table olives at different salt concentrations (4, 5, 6, 8%) trough experimental (E) and spontaneous (C) fermentation (1 to 120 days).

**DAYS OF FERMENTATION**							
	**1**	**7**	**15**	**30**	**60**	**90**	**120**
**MESOPHILIC BACTERIA**							
	7.36 ± 0.05^d^	7.75 ± 0.06^g^	7.53 ± 0.20^e^	7.74 ± 0.05^e^	7.44 ± 0.18^d^	7.15 ± 0.12^d^	7.35 ± 0.08^f^
E1							
E2	7.31 ± 0.06^d^	7.25 ± 0.30^f^	7.51 ± 0.06^e^	7.01 ± 0.12^d^	7.40 ± 0.10^d^	7.11 ± 0.14^d^	7.01 ± 0.08^ef^
E3	7.23 ± 0.09^d^	6.95 ± 0.43^e^	7.49 ± 0.06^e^	6.99 ± 0.02^d^	6.65 ± 0.09^c^	6.93 ± 0.10^d^	7.05 ± 0.14^e^
E4	6.56 ± 0.11^c^	6.75 ± 0.18^d^	6.53 ± 0.24^d^	5.94 ± 0.41^c^	5.12 ± 0.37^a^	5.82 ± 0.24^b^	5.73 ± 0.13^c^
C1	4.21 ± 0.06^a^	4.40 ± 0.21^a^	4.83 ± 0.33^a^	5.20 ± 0.09^a^	5.11 ± 0.11^a^	5.36 ± 0.08^a^	5.07 ± 0.05^a^
C2	4.38 ± 0.10^a^	4.53 ± 0.15^ab^	5.02 ± 0.21^b^	5.92 ± 0.09^c^	5.36 ± 0.07^ab^	5.60 ± 0.06^bc^	5.39 ± 0.06^b^
C3	4.89 ± 0.09^b^	4.81 ± 0.05^ac^	5.51 ± 0.18^c^	5.97 ± 0.11^c^	5.45 ± 0.09^ab^	5.40 ± 0.12^ac^	5.53 ± 0.13^bc^
C4	4.82 ± 0.03^b^	4.68 ± 0.18^a^	5.21 ± 0.21^b^	5.68 ± 0.28^b^	5.63 ± 0.12^b^	5.58 ± 0.23^ac^	5.74 ± 0.12^cd^
**LACTIC ACID BACTERIA**							
	7.09 ± 0.10^d^	7.52 ± 0.12^g^	8.08 ± 0.25^g^	8.72 ± 0.15^f^	8.13 ± 0.12^e^	7.85 ± 0.13^d^	7.83 ± 0.07^de^
E1							
E2	7.12 ± 0.10^de^	7.59 ± 0.13^g^	7.38 ± 0.30^d^	8.27 ± 0.07^e^	8.49 ± 0.05^d^	8.58 ± 0.08^e^	8.23 ± 0.11^d^
E3	7.02 ± 0.21^de^	7.14 ± 0.05^f^	7.15 ± 0.35^de^	7.03 ± 0.10^b^	7.64 ± 0.07^c^	7.86 ± 0.11^d^	7.79 ± 0.15^de^
E4	7.03 ± 0.11^e^	7.37 ± 0.27^e^	7.34 ± 0.09^df^	7.02 ± 0.21^abc^	7.30 ± 0.40^b^	7.05 ± 0.53^c^	7.04 ± 0.30^c^
C1	4.51 ± 0.20^abc^	4.69 ± 0.13^b^	5.81 ± 0.05^c^	7.36 ± 0.11^cd^	7.83 ± 0.05^c^	6.88 ± 0.13^b^	6.00 ± 0.08^a^
C2	4.48 ± 0.23^c^	4.79 ± 0.18^bc^	5.96 ± 0.07^c^	7.21 ± 0.07^ad^	7.32 ± 0.06^b^	6.04 ± 0.09^a^	5.96 ± 0.11^a^
C3	4.21 ± 0.16^b^	4.57 ± 0.09^bd^	5.53 ± 0.13^b^	7.10 ± 0.13^ad^	6.74 ± 0.12^a^	6.78 ± 0.10^b^	5.67 ± 0.06^a^
C4	3.93 ± 0.21^a^	3.85 ± 0.09^a^	5.12 ± 0.21^a^	6.95 ± 0.35^a^	6.58 ± 0.14^a^	5.96 ± 0.42^a^	5.94 ± 0.36^a^
**YEASTS**							
	4.00 ± 0.01^d^	4.63 ± 0.13^c^	5.27 ± 0.28^a^	5.13 ± 0.06^a^	5.47 ± 0.18^a^	5.20 ± 0.12^ab^	4.61 ± 0.05^b^
E1							
E2	3.97 ± 0.10^c^	4.18 ± 0.06^b^	5.35 ± 0.31^a^	5.61 ± 0.09^a^	5.56 ± 0.11^a^	5.60 ± 0.15^b^	4.11 ± 0.06^a^
E3	3.84 ± 0.01^a^	5.05 ± 0.14^d^	5.62 ± 0.31^ab^	5.28 ± 0.07^a^	5.51 ± 0.10^a^	5.14 ± 0.11^a^	4.30 ± 0.05^ab^
E4	3.93 ± 0.21^b^	3.46 ± 0.11^a^	5.81 ± 0.18^b^	5.23 ± 0.21^a^	5.68 ± 0.30^a^	5.12 ± 0.24^a^	5.50 ± 0.23^c^
C1	4.21 ± 0.12^g^	5.34 ± 0.30^d^	6.73 ± 0.18^c^	6.25 ± 0.12^b^	6.28 ± 0.10^b^	5.32 ± 0.09^ab^	5.58 ± 0.09^c^
C2	4.28 ± 0.23^bcd^	6.12 ± 0.32^e^	6.61 ± 0.21^c^	6.85 ± 0.11^c^	6.48 ± 0.11^bc^	5.28 ± 0.14^ab^	5.72 ± 0.08^c^
C3	4.09 ± 0.09^e^	5.03 ± 0.11^d^	5.61 ± 0.11^ab^	6.16 ± 0.08^b^	5.47 ± 0.05^ab^	5.00 ± 0.09^a^	5.36 ± 0.14^c^
C4	4.14 ± 0.23^f^	4.96 ± 0.16^cd^	5.78 ± 0.23^ab^	5.63 ± 0.31^a^	5.91 ± 0.26^ab^	5.32 ± 0.27^ab^	5.40 ± 0.11^c^
**STAPHYLOCOCCI**							
	4.76 ± 0.08^b^	4.23 ± 0.14^a^	4.72 ± 0.21^bc^	4.83 ± 0.26^b^	4.79 ± 0.15^ab^	4.19 ± 0.18^a^	3.30 ± 0.23^ac^
E1							
E2	4.18 ± 0.32^a^	4.42 ± 0.33^a^	4.97 ± 0.35^cd^	4.12 ± 0.15^a^	4.65 ± 0.07^a^	4.44 ± 0.23^ac^	3.72 ± 0.32^bcd^
E3	4.10 ± 0.16^a^	4.33 ± 0.18^a^	4.54 ± 0.15^ab^	4.21 ± 0.31^a^	4.64 ± 0.09^a^	4.93 ± 0.21^bd^	3.14 ± 0.20^a^
E4	4.03 ± 0.21^a^	4.45 ± 0.23^a^	4.26 ± 0.32^a^	4.83 ± 0.21^bc^	4.62 ± 0.26^ab^	4.30 ± 0.21^ac^	3.04 ± 0.18^a^
C1	4.81 ± 0.14^b^	5.18 ± 0.11^b^	5.44 ± 0.11^ef^	5.18 ± 0.08^bc^	5.18 ± 0.12^ac^	4.62 ± 0.05^acd^	3.58 ± 0.11^c^
C2	4.76 ± 0.18^ab^	5.05 ± 0.19^b^	5.73 ± 0.21^f^	5.24 ± 0.18^c^	5.11 ± 0.15^a^	4.74 ± 0.10^bcd^	3.74 ± 0.14^cd^
C3	4.27 ± 0.11^a^	5.04 ± 0.09^b^	5.53 ± 0.09^ef^	4.72 ± 0.06^b^	4.98 ± 0.10^a^	4.63 ± 0.06^bcd^	3.96 ± 0.07^d^
C4	4.34 ± 0.12^a^	5.08 ± 0.30^b^	5.11 ± 0.12^de^	4.77 ± 0.26^b^	4.73 ± 0.34^a^	4.23 ± 0.31^ac^	3.82 ± 0.31^cd^
**ENTEROBACTERIA**							
	2.99 ± 0.24^b^	2.33 ± 0.28^b^	2.40 ± 0.18^cd^	1.03 ± 0.11^a^		<1	<1
E1					<1		
E2	2.24 ± 0.06^a^	2.13 ± 0.14^bc^	1.09 ± 0.10^a^	<1	<1	<1	<1
E3	2.00 ± 0.13^a^	2.26 ± 0.09^bc^	1.84 ± 0.16^c^	1.32 ± 0.13^a^	1.05 ± 0.21^a^	<1	<1
E4	2.19 ± 0.07^a^	1.74 ± 0.21^ac^	1.45 ± 0.23^b^	1.19 ± 0.21^a^	1.02 ± 0.07^a^	<1	<1
C1	3.91 ± 0.09^c^	4.11 ± 0.15^d^	3.53 ± 0.15^e^	2.37 ± 0.24^b^	2.32 ± 0.11^b^	1.63 ± 0.13^b^	1.14 ± 0.05^a^
C2	3.82 ± 0.13^c^	4.02 ± 0.21^dg^	3.13 ± 0.09^de^	2.50 ± 0.18^b^	2.67 ± 0.15^bc^	1.84 ± 0.21^b^	1.45 ± 0.12^ab^
C3	3.89 ± 0.18^c^	3.97 ± 0.12^df^	3.23 ± 0.18^e^	2.15 ± 0.09^b^	2.31 ± 0.13^b^	1.54 ± 0.18^b^	1.55 ± 0.08^b^
C4	3.98 ± 0.12^c^	3.31 ± 0.23^de^	3.64 ± 0.14^e^	2.06 ± 0.09^b^	2.09 ± 0.21^bd^	1.14 ± 0.13^a^	1.39 ± 0.03^ab^

*a–g for each medium data in the same column with different superscript letters are significantly different (P < 0.05)*.

### Volatile organic compound (VOC) detection by gas chromatography-mass-spectrometry (GC-MS)

**Figure 3:**
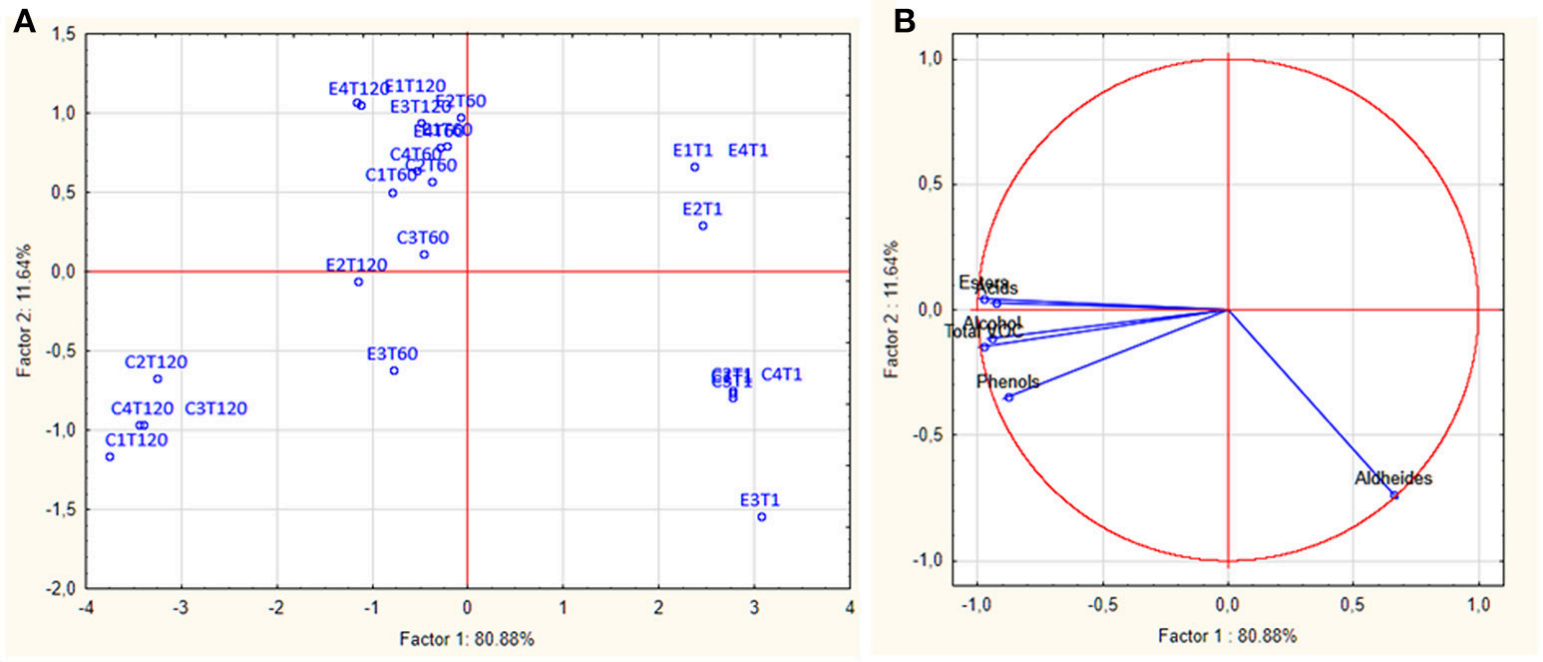
PCA plot **(A)** and score plot **(B)** showing the distribution of experimental (E) and control (C) samples through the fermentation process.

Volatile organic compounds (VOCs) of E and C brine samples at 1, 60, and 120 d of fermentation, are reported in Table 2. The assessment allowed the identification of 47 compounds as acids, alcohols, esters, aldehydes, and phenols. Overall, total VOCs exhibited a growing trend during the fermentation reaching an average value of 2349.70 μg/l after 120 days. In particular, the highest values were registered in all control samples, with a mean value of 3215.23 μg/l. In detail, in all samples, at beginning of fermentation, aldehydes represented the main VOCs, and after 60 days, they significantly decreased, whereas alcohols, acids, esters and phenols increased. At the end of fermentation (120 d), differences were observed among brine samples. Zooming on each chemical class, it is possible to assert that overall, the detected amounts of each compounds were sample-dependent. Among acids, the acetic acid was the most abundant compound, with the highest values in control samples, whereas hexanoic and propionic acids were more abundant in experimental samples (Table 2). Among alcohols, ethanol dominated the fermentation process, especially in control samples, followed by isoamyl- and phenyl-ethyl alcohol. Among esters, the highest amount was achieved by ethyl-acetate, followed by ethyl lactate. A different trend was revealed for butanoic-acid-2-methylester, which showed the highest value in the E2 sample, followed by control samples. The most abundant aldehyde and phenols were nonanal and cresol, respectively. Evaluating the VOCs occurrence percentage on E and C samples at 120 days of fermentation (Figure S1), it is interesting to note that esters and acids were mainly present in all E samples with the highest occurrence percentage in E2 (31.7 and 11.3%, respectively). Alcohols, phenols, and aldehydes were also detected at high occurrence percentage in E2 sample (Figure S1). Figures 3A,B showed the PCA plot of distribution of C (C1-C4) and E (E1-E4) samples, at different days of fermentation, in the PC1–PC2 plane. Based on the loadings (data not shown), component 1, that represent the 80.88% of the variability, can be viewed as an esters factor, while the second principal component (variance 11.64%), was mainly represented by aldehydes. Score plots are effective in showing the difference among samples and in separating them in the graphs. In detail, all E and C samples at 1 day of fermentation highlighted a positive contribution by component 2 planes. After 120 d, C samples were characterized by a positive contribution of alcohols whereas E samples principally by esters.

**Table 2:** Volatile organic compounds (VOCs) expressed as μg/l of Nocellara Etnea brine samples at different salt concentrations (4, 5, 6, 8%) under controlled (E) and spontaneous fermentation (C) at 1, 60, and 120 days.

		**E1**			**E2**			**E3**			**E4**			**C1**			**C2**			**C3**			**C4**		
	**RT**	**1**	**60**	**120**	**1**	**60**	**120**	**1**	**60**	**120**	**1**	**60**	**120**	**1**	**60**	**120**	**1**	**60**	**120**	**1**	**60**	**120**	**1**	**60**	**120**
**Acids**		**0.00**	**142.29**	**113.26**	**0.00**	**69.69**	**154.59**	**0.00**	**69.55**	**108.06**	**0.00**	**75.84**	**107.60**	**0.00**	**69.52**	**139.04**	**0.00**	**71.35**	**136.14**	**0.00**	**60.25**	**134.61**	**0.00**	**59.23**	**131.78**
Acetic acid	24.78	0.00	66.75^bc^	63.24^b^	0.00	45.06^a^	81.84^d^	0.00	42.76^a^	58.34^b^	0.00	48.01^a^	60.21^b^	0.00	69.52^c^	108.83^e^	0.00	70.23^c^	105.24^e^	0.00	60.25^b^	103.91*e*	0.00	59.23^b^	102.85^e^
Propionic acid	29.74	0.00	4.02^c^	9.08	0.00	5.38^d^	31.23^e^	0.00	1.03^b^	0.00	0.00	1.02^b^	0.09^a^	0.00	0.00	3.00^c^	0.00	0.00	2.85^c^	0.00	0.00	3.15^c^	0.00	0.00	3.13^c^
Isobutyric acid	31.43	0.00	8.96^f^	7.01^e^	0.00	1.26^a^	3.04^c^	0.00	5.39^d^	1.67^a^	0.00	5.65^d^	1.88^ab^	0.00	0.00	2.12^ab^	0.00	1.12^a^	2.64^c^	0.00	0.00	2.36^c^	0.00	0.00	2.29^b^
Butanoic acid	35.01	0.00	2.31^e^	1.78^d^	0.00	0.94^c^	4.51^f^	0.00	0.59^b^	1.52^d^	0.00	0.89^c^	1.01^c^	0.00	0.00	0.25^a^	0.00	0.00	0.23^a^	0.00	0.00	0.95^c^	0.00	0.00	0.86^c^
Hexanoic acid	36.19	0.00	0.00	30.12^e^	0.00	7.93^a^	32.98^e^	0.00	16.54^cd^	45.34^f^	0.00	17.23^d^	43.21^f^	0.00	0.00	15.21^c^	0.00	0.00	14.21^c^	0.00	0.00	12.56^b^	0.00	0.00	11.99^b^
2-Ethylheptanoic acid	57.20	0.00	60.25^d^	2.03^a^	0.00	9.12^c^	0.99^a^	0.00	3.24^a^	1.19^a^	0.00	3.04^a^	1.20^a^	0.00	0.00	9.63^b^	0.00	0.00	10.97^b^	0.00	0.00	11.68^b^	0.00	0.00	10.66^b^
**Alcohol**		**19.20**	**632.36**	**434.45**	**48.32**	**548.94**	**449.10**	**29.40**	**1041.55**	**1082.59**	**30.01**	**1047.93**	**1084.68**	**67.71**	**1056.92**	**2165.14**	**61.46**	**1048.11**	**2019.43**	**69.49**	**1028.98**	**1966.09**	**64.05**	**937.38**	**1961.51**
Ethanol	3.33	0.00	250.14^ab^	236.95^ab^	0.00	350.21^b^	165.24^a^	0.00	736.45^c^	768.00^c^	0.00	735.84^c^	769.12^c^	0.00	705.91^c^	1754.23^d^	0.00	694.81^c^	1613.28^d^	0.00	681.21^c^	1570.21^d^	0.00	601.25^c^	1568.91^d^
Isoamylalcohol	11.58	5.95^a^	81.25^c^	60.01^b^	0.00	59.11^b^	71.98^c^	2.81^a^	65.34^b^	88.28^c^	2.89^a^	65.21^b^	87.56^c^	0.00	84.87^c^	172.13^d^	0.00	83.14^c^	169.87^d^	0.00	79.56^c^	160.85^d^	0.00	79.11^c^	159.27^d^
1-Hexanol	19.23	1.03^a^	9.08^b^	11.56^c^	0.00	13.03^cd^	14.74^d^	0.00	12.11^c^	11.37^c^	0.00	13.84^d^	11.59^c^	0.00	15.21^d^	18.25^e^	0.00	12.24^c^	18.12^e^	0.00	16.02^d^	16.59^d^	0.00	14.96^d^	18.07^e^
cis Hexen 1 ol	20.84	0.00	81.65^c^	41.21^a^	0.00	35.83^a^	30.98^a^	0.00	67.32^b^	55.41^b^	0.00	67.89^b^	54.98^b^	0.00	61.23^b^	88.46^c^	0.00	62.29^b^	88.23^c^	0.00	61.21^b^	87.69^c^	0.00	58.41^b^	86.30^c^
3-Octenol	25.13	0.00	1.96^c^	2.58^de^	0.00	0.99^a^	2.01^c^	0.00	2.45^d^	1.69^b^	0.00	2.39^cd^	1.72^b^	0.00	1.20^a^	3.02^e^	0.00	2.03^c^	2.91^e^	0.00	1.45^ab^	3.01^e^	0.00	1.11^a^	2.47^d^
1-Heptanol	25.79	2.36^a^	9.02^f^	3.99^c^	3.01^b^	4.96^d^	5.12^d^	2.07^a^	5.34^d^	2.79^ab^	2.05^a^	5.58^d^	2.81^ab^	3.45^bc^	9.52^f^	6.13^e^	3.25^b^	13.21^g^	6.09^e^	3.56^bc^	11.23^g^	5.99^de^	3.23^b^	9.98^f^	5.59^d^
1-Octanol	31.12	0.00	21.96^d^	2.46^a^	14.89^c^	2.78^a^	12.56^c^	13.12^c^	11.03^c^	8.42^b^	12.97^c^	12.01^c^	9.01^b^	20.31^d^	21.23^d^	4.01^a^	20.28^d^	21.43^d^	3.95^a^	21.59^d^	24.47^d^	3.54^a^	20.85^d^	22.83^d^	3.48^a^
1-Nonanol	35.91	8.74^a^	68.32^c^	0.00	5.87^a^	0.00	0.00	4.02^a^	0.00	0.00	4.65^a^	0.00	0.00	4.99^a^	27.14^b^	0.00	4.85^a^	26.93^b^	0.00	5.80^a^	24.61^b^	0.00	5.01^a^	23.74^b^	0.00
Benzyl Alcohol	47.77	0.00	43.21^e^	0.00	0.00	9.18^a^	16.35^b^	0.00	29.06^d^	0.00	0.00	30.15^d^	0.00	0.00	21.93^c^	0.00	0.00	18.36^c^	0.00	0.00	20.25^c^	0.00	0.00	19.26^c^	0.00
Phenylethyl alcohol	50.96	0.00	62.35^a^	75.69^s^	0.00	72.85^a^	130.12^c^	0.00	112.45^b^	146.62^c^	0.00	115.02^b^	147.89^c^	0.00	105.47^b^	118.91^b^	0.00	110.29^b^	116.98^b^	0.00	105.26^b^	118.21^b^	0.00	103.61^b^	117.42^b^
1-Undecanol	53.54	1.12^a^	1.59^a^	0.00	21.92^d^	0.00	0.00	7.38^c^	0.00	0.00	7.45^c^	0.00	0.00	36.27^e^	3.21^b^	0.00	31.22^e^	3.38^b^	0.00	36.44^e^	3.71^b^	0.00	32.98^e^	3.12^b^	0.00
1-Dodecanol	63.35	0.00	1.83^a^	0.00	2.63^b^	0.00	0.00	0.00	0.00	0.00	0.00	0.00	0.00	2.69^b^	0.00	0.00	1.86^a^	0.00	0.00	2.10^a^	0.00	0.00	1.98^a^	0.00	0.00
**Esters**		**0.00**	**392.42**	**287.06**	**0.00**	**287.25**	**434.10**	**0.00**	**346.49**	**490.94**	**0.00**	**356.58**	**480.07**	**0.00**	**379.66**	**626.99**	**0.00**	**247.02**	**458.30**	**0.00**	**375.88**	**641.68**	**0.00**	**378.62**	**640.34**
Ethyl acetate	2.75	0.00	126.38^a^	127.54^a^	0.00	127.91^a^	150.52^a^	0.00	134.65^a^	197.56^ab^	0.00	140.83^a^	189.12^ab^	0.00	201.30^b^	347.21^c^	0.00	154.28^a^	256.81^bc^	0.00	202.70^b^	352.42^c^	0.00	205.61^b^	350.14^c^
Ethyl propanoate	3.56	0.00	0.00	0.00	0.00	0.00	7.12	0.00	0.00	0.00	0.00	0.00	0.00	0.00	0.00	0.00	0.00	0.00	0.00	0.00	0.00	0.00	0.00	0.00	0.00
Ethyl butanoate	4.07	0.00	0.00	0.00	0.00	1.87^a^	8.96^c^	0.00	2.30^a^	10.45^d^	0.00	2.19^a^	11.02^d^	0.00	8.86^c^	0.00	0.00	4.56^b^	0.00	0.00	9.86^cd^	0.00	0.00	8.88^c^	0.00
Butanoic acid 2 methylester	5.44	0.00	6.53^a^	11.04^b^	0.00	14.76^b^	93.11^f^	0.00	5.67^a^	11.98^b^	0.00	6.01^a^	12.86^b^	0.00	24.39^c^	69.23^e^	0.00	13.32^b^	62.13^d^	0.00	23.62^c^	74.37^e^	0.00	25.84^c^	72.86^e^
Butanoic acid 3 methylester	5.85	0.00	2.86^a^	5.42^b^	0.00	12.90^d^	65.01^f^	0.00	4.34^b^	17.63^e^	0.00	4.16^b^	17.84^e^	0.00	2.51^a^	9.89^c^	0.00	1.23^a^	9.61^c^	0.00	2.84^a^	9.56^c^	0.00	2.51^a^	9.99^c^
Isoamylacetate	7.47	0.00	2.69^a^	16.31^b^	0.00	9.31^b^	11.82^b^	0.00	4.53^a^	14.11^b^	0.00	4.86^a^	14.82^b^	0.00	3.22^a^	45.23^c^	0.00	1.75^a^	42.18^c^	0.00	3.65^a^	48.51^c^	0.00	3.46^a^	47.02^c^
Hexanoic acid methylester	9.90	0.00	0.00	2.18	0.00	0.00	0.00	0.00	0.00	0.00	0.00	0.00	0.00	0.00	0.00	0.00	0.00	0.00	0.00	0.00	0.00	0.00	0.00	0.00	0.00
Ethyl hexanoate	12.29	0.00	16.89^d^	13.02^cd^	0.00	8.75^b^	3.99^a^	0.00	11.09^c^	18.34^d^	0.00	11.21^c^	19.05^d^	0.00	9.06^b^	3.98^a^	0.00	4.56^a^	3.21^a^	0.00	9.17^b^	4.57^a^	0.00	9.01^b^	4.32^a^
Ethyl lactate	18.49	0.00	61.53^e^	56.25^e^	0.00	34.39^c^	42.98^d^	0.00	42.34^d^	80.67^f^	0.00	42.76^d^	81.12^f^	0.00	23.95^b^	51.74^e^	0.00	11.98^a^	32.12^c^	0.00	22.86^b^	45.79^d^	0.00	23.12^b^	49.56^de^
Butanoic acid 2-hydroxy-3-methylester	23.10	0.00	4.02^a^	4.41^a^	0.00	0.00	4.01^a^	0.00	0.00	3.40^a^	0.00	0.00	3.98^a^	0.00	0.00	7.98^c^	0.00	0.00	6.36^b^	0.00	0.00	8.82^c^	0.00	0.00	8.17^c^
Ethyl octanoate	23.44	0.00	9.63^d^	1.84^b^	0.00	1.78^b^	0.34^a^	0.00	2.66^bc^	0.32^a^	0.00	2.97^c^	0.56^a^	0.00	8.99^d^	1.82^b^	0.00	1.50^b^	0.96^ab^	0.00	9.61^d^	1.38^b^	0.00	9.09^d^	1.79^b^
Ethyl-3-hydroxybutyrate	28.92	0.00	10.27^e^	7.08^d^	0.00	11.15^e^	7.21^d^	0.00	2.98^c^	0.00	0.00	2.81^c^	0.00	0.00	8.60^de^	1.97^b^	0.00	0.00	0.89^a^	0.00	0.00	1.49^ab^	0.00	0.00	1.89^b^
Pentanoic acid 2-hydroxy-4-methyl-ethylester	30.00	0.00	16.83^f^	7.21^c^	0.00	5.16^b^	7.85^c^	0.00	10.45^d^	12.49^e^	0.00	9.99^d^	13.15^e^	0.00	6.92^c^	15.19^e^	0.00	3.69^a^	7.21^c^	0.00	6.78^c^	15.53^e^	0.00	6.77^c^	15.15^e^
Decanoic acid methylester	32.95	0.00	4.10^c^	2.00^b^	0.00	0.00	0.00	0.00	0.00	0.00	0.00	0.00	0.00	0.00	0.00	0.52^a^	0.00	0.00	0.00	0.00	0.00	0.68^a^	0.00	0.00	0.49^a^
Ethyl decanoate	35.77	0.00	5.18^c^	1.74^b^	0.00	1.42^b^	0.36^a^	0.00	1.45^b^	1.46^b^	0.00	1.24^b^	1.51^b^	0.00	1.74^b^	5.01^c^	0.00	1.79^b^	2.38^b^	0.00	1.96^b^	4.78^c^	0.00	1.86^b^	5.02^c^
Ethyl benzoate	36.74	0.00	5.30^bc^	4.81^b^	0.00	12.38^d^	3.86^b^	0.00	3.89^b^	5.87^c^	0.00	3.92^b^	5.71^bc^	0.00	2.45^a^	5.63^c^	0.00	1.54^a^	1.99^a^	0.00	2.71^a^	5.29^bc^	0.00	2.41^a^	5.86^c^
eAcetic acid, 2 phenylethyl	43.01	0.00	3.04^c^	1.83^b^	0.00	8.72^d^	2.31^b^	0.00	0.99^a^	2.54^bc^	0.00	1.05^a^	2.45^bc^	0.00	1.65^b^	3.98^c^	0.00	0.96^a^	1.75^b^	0.00	1.85^b^	3.99^c^	0.00	1.72^b^	4.14^c^
Methyl Hydrocinnamate	45.91	0.00	19.98^d^	4.89^b^	0.00	0.00	7.16^bc^	0.00	10.34^c^	13.69^d^	0.00	10.58^c^	13.63^d^	0.00	10.69^c^	4.82^b^	0.00	5.65^b^	2.13^a^	0.00	10.74^c^	4.87^b^	0.00	10.36^c^	4.39^b^
Ethyl dodecanoate	46.20	0.00	15.03^bc^	0.30^a^	0.00	9.63^b^	0.51^a^	0.00	10.45^b^	1.32^a^	0.00	10.75^b^	1.41^a^	0.00	1.06^a^	20.23^c^	0.00	0.80^a^	8.96^b^	0.00	1.32^a^	20.64^c^	0.00	1.12^a^	21.03^c^
Ethyl Hydrocinnamate	48.97	0.00	82.16^d^	19.19*a*	0.00	27.12^ab^	16.98^a^	0.00	98.36^d^	99.11^d^	0.00	101.25^e^	91.84^de^	0.00	64.31^c^	32.59^b^	0.00	39.41^b^	19.61^a^	0.00	66.21^c^	38.99^b^	0.00	66.86^c^	38.52^b^
**Aldehides**		**213.48**	**113.96**	**28.62**	**104.29**	**32.79**	**75.99**	**82.71**	**55.02**	**31.62**	**82.85**	**55.29**	**32.38**	**166.44**	**46.50**	**76.45**	**165.87**	**48.18**	**52.66**	**168.02**	**74.49**	**75.31**	**165.79**	**44.82**	**75.50**
Octanal	14.81	19.20^e^	19.85^e^	2.85^a^	11.85^d^	4.17^b^	5.89^bc^	8.34^c^	13.99^d^	4.63^b^	8.17^c^	14.02^de^	4.83^b^	17.12^e^	6.09^bc^	10.23^d^	17.45^e^	7.52^c^	5.21^b^	16.95^e^	12.28^d^	9.96^d^	16.98^e^	5.48^b^	10.32^d^
Nonanal	20.78	86.98^g^	27.01^d^	11.98^a^	43.92^e^	11.01^a^	32.18^d^	37.23^de^	20.25^c^	10.43^a^	37.41^de^	20.28^c^	10.79^a^	71.23^f^	15.21^b^	25.96^cd^	70.84^f^	15.48^b^	19.85^c^	69.82^f^	32.11^d^	25.63^cd^	70.94^f^	15.02^b^	26.41^cd^
3-Octanal	21.64	0.00	2.04^a^	5.21^b^	0.00	1.74^a^	1.89^a^	0.00	2.05^a^	0.00	0.00	2.10^a^	0.00	0.00	2.21^a^	2.49^a^	0.00	2.15^a^	2.56^a^	0.00	2.01^a^	2.06^a^	0.00	2.07^a^	2.30^a^
Decanal	26.85	107.30^h^	41.21^ef^	4.56^a^	47.52^f^	6.87^b^	21.02^d^	35.74^e^	6.45^b^	4.91^a^	35.76^e^	6.58^b^	5.01^a^	73.24^g^	10.85^c^	21.56^d^	72.61^g^	10.78^c^	10.11^c^	76.23^g^	16.57^cd^	21.54^d^	73.01^g^	10.20^c^	19.99^d^
Benzaldehyde	28.08	0.00	23.85^e^	4.02^b^	1.00^a^	9.00^c^	15.01^d^	1.40^a^	12.27^c^	11.65^c^	1.51^a^	12.31^c^	11.75^c^	4.85^b^	12.14^c^	16.21^d^	4.96^b^	12.25^c^	14.93^d^	5.02^b^	11.52^c^	16.12^d^	4.86^b^	12.05^c^	16.48^d^
**Phenols**		**0.00**	**202.76**	**181.60**	**0.00**	**129.26**	**255.00**	**0.00**	**9.85**	**52.39**	**0.00**	**9.99**	**52.58**	**0.00**	**148.47**	**412.65**	**0.00**	**116.02**	**395.65**	**0.00**	**123.91**	**376.73**	**0.00**	**126.67**	**374.95**
Guaiacol	47.25	0.00	16.93^a^	0.00	0.00	0.00	31.08^b^	0.00	0.00	11.84^a^	0.00	0.00	12.01^a^	0.00	61.02^c^	70.25^d^	0.00	57.16^c^	63.55^c^	0.00	64.13^c^	54.23^c^	0.00	66.84^cd^	55.01^c^
Creosol (Homoguaiacol)	52.57	0.00	185.23^f^	175.28^f^	0.00	116.01^e^	205.83^f^	0.00	3.65^a^	32.57^c^	0.00	3.89^a^	32.41^c^	0.00	58.62^d^	301.25^g^	0.00	17.89^b^	292.14^g^	0.00	18.30^b^	284.60^g^	0.00	18.76^b^	281.52^g^
Phenol	55.36	0.00	0.60^a^	1.96^a^	0.00	1.41^c^	4.02^b^	0.00	0.59^a^	1.01^a^	0.00	0.63^a^	1.04^a^	0.00	6.98^bc^	10.21^c^	0.00	5.69^b^	9.81^c^	0.00	5.12^b^	9.78^c^	0.00	5.05^b^	9.46^c^
4-Ethyl phenol	63.20	0.00	0.00	4.36^a^	0.00	11.84^b^	14.07^b^	0.00	5.61^a^	6.97^a^	0.00	5.47^a^	7.12^a^	0.00	21.85^c^	30.94^d^	0.00	35.28^e^	30.15^d^	0.00	36.36^e^	28.12^d^	0.00	36.02^e^	28.96^d^
**Total**		**232.68**	**1483.79**	**1044.99**	**152.61**	**1067.93**	**1368.78**	**112.11**	**1522.46**	**1765.60**	**112.86**	**1545.63**	**1757.31**	**234.15**	**1701.07**	**3420.27**	**227.33**	**1530.68**	**3062.18**	**237.51**	**1663.51**	**3194.42**	**229.84**	**1546.72**	**3184.08**

*a–g for each compound different superscript letters in the same raw are significantly different (P < 0.05)*.

### Permutation analysis and correlations between microbiota and metabolome of nocellara etnea table olives

**Figure 4:**
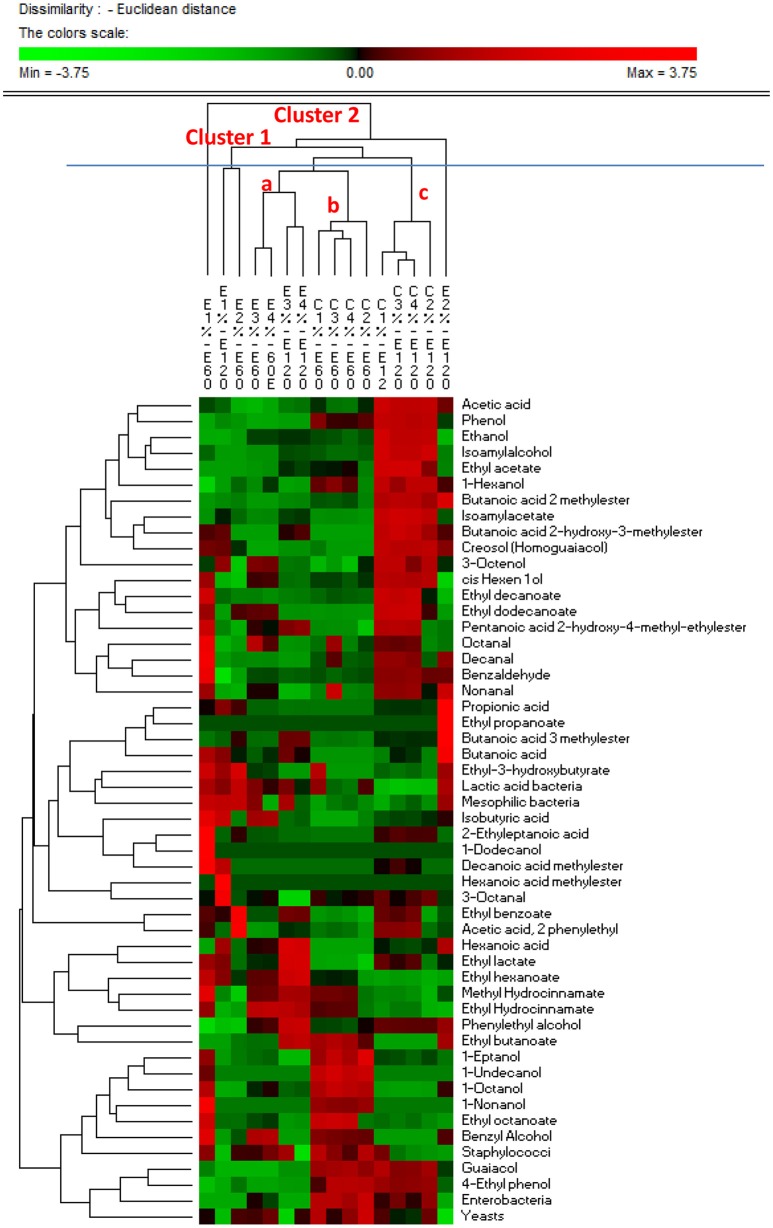
PermutMatrixEN analysis of the microbial and metabolomics profiles of experimental (E) and control (C) Nocellara Etnea under different salt concentrations (4, 5, 6, 8%) at 60 and 120 days.

Similarities in the observed microbial counts and metabolomics profiles between samples at 60 and 120 days of fermentation were estimated using the PermutMatrixEN software (Figure 4). In detail, two clusters were revealed, showing that overall the samples grouped based on the addition of starters and on time of the fermentation. It is interesting to note that salt content did not discriminate samples, with the exception of the experimental samples E1 and E2 at 60 and 120 d, respectively, which exhibited unique profiles. In fact, these samples showed the most divergent microbial and metabolic profiles, with a strong presence of ethyl-3-hydroxybutyrate, LAB, mesophilic bacteria, isobutyric acid, 2-ethyleptanoic acid, 1-dodecanol, decanoic acid methyl ester, methyl idrocynnamate, 1-octanol, 1-nonanol, ethyl octanoate, benzyl alcohol, staphylococci, octanal, decanal, benzaldehyde, nonanal in the E1 sample at 60 days, and of butanoic acid 2 methylester, propionic acid, ethyl propanoate, butanoic acid 3 methylester, hexanoic acid, phenylethyl alcohol, ethyl butanoate in the E2 sample at 120 days. Compared to 60 days of fermentation, control samples at 120 days showed different microbial and metabolomics profiles with a strong increase in acetic acid, phenol, ethanol, isoamyl alcohol, ethyl acetate, 1-hexanol, isoamylacetate, butanoic acid 2-hydroxy-3-methylester, creosol (homoguaiacol), 3-octenol, cis hexen 1 ol, ethyl decanoate, ethyl dodecanoate, and nonanal. Evaluating E samples, both, time of fermentation and NaCl content, affected the microbial and metabolomics profiles. High similarity was found between samples treated with 6 and 8% of NaCl after 60 and 120 d of fermentation, characterized by a lower amount of guaiacol, 4-ethyl phenol and enterobacteria.

**Figure 5:**
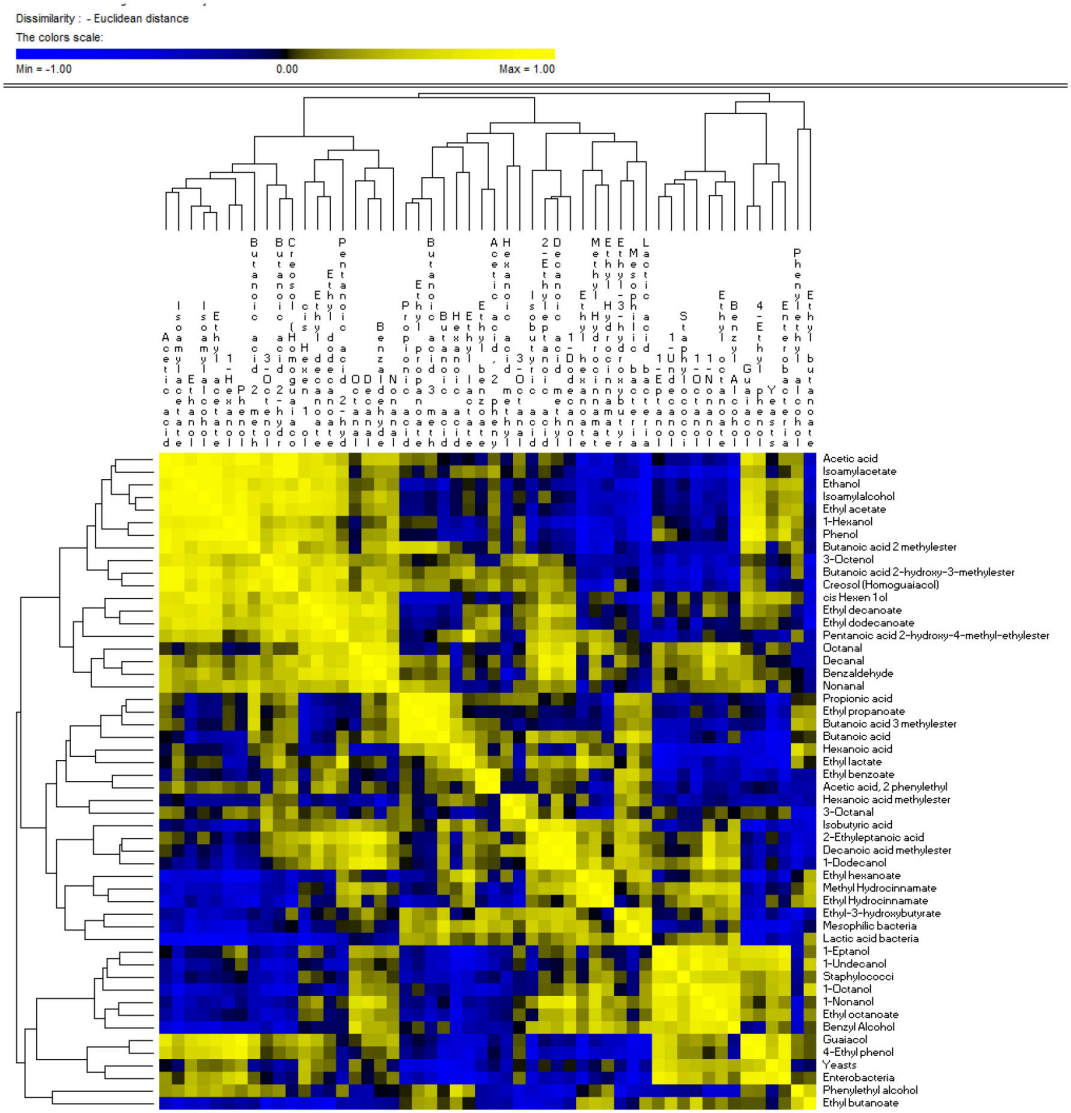
Significant correlations between microbial and metabolomics data found after 60 and 120 days of fermentation of experimental (E) and control (C) Nocellara Etnea under different salt concentrations (4, 5, 6, 8%).

Correlations between microbial and metabolomics data are shown in Figure 5. Among organic acids, propionic, isobutyric acid, butanoic, and exanoic compounds were negatively correlated with yeasts and staphylococci, and positively correlated (*r* > 0.30, *p* < 0.05) with mesophilic bacteria. One exception was the acetic acid, which, was positively correlated (*r* = 0.238, *p* = 0.045) with enterobacteria and negatively with LAB (*r* = −0.735; *p* = 0.048). Zooming on the metabolomics, the acetic acid was positively correlated (*r* > 0.70, *p* < 0.05) with alcohols (ethanol, isoamylalcohol,1-hexanol, cis hexen 1 ol and 3-octenol) and esters (ethyl acetate, butanoic acid 2 methylester, isoamylacetate, butanoic acid, and 2-hydroxy-3-methylester). Alcohols, with the exception of 3-octenol, phenylethyl alcohol and 1-dodecanol, were positively correlated with enterobacteria and yeasts. In addition, ethanol, isoamylalcohol, 1-hexanol, cis hexen 1 ol, and 3-octenol were negatively correlated with mesophilic bacteria and LAB.

### Molecular identification of *Lactobacillus* spp. isolates

**Figure 6:**
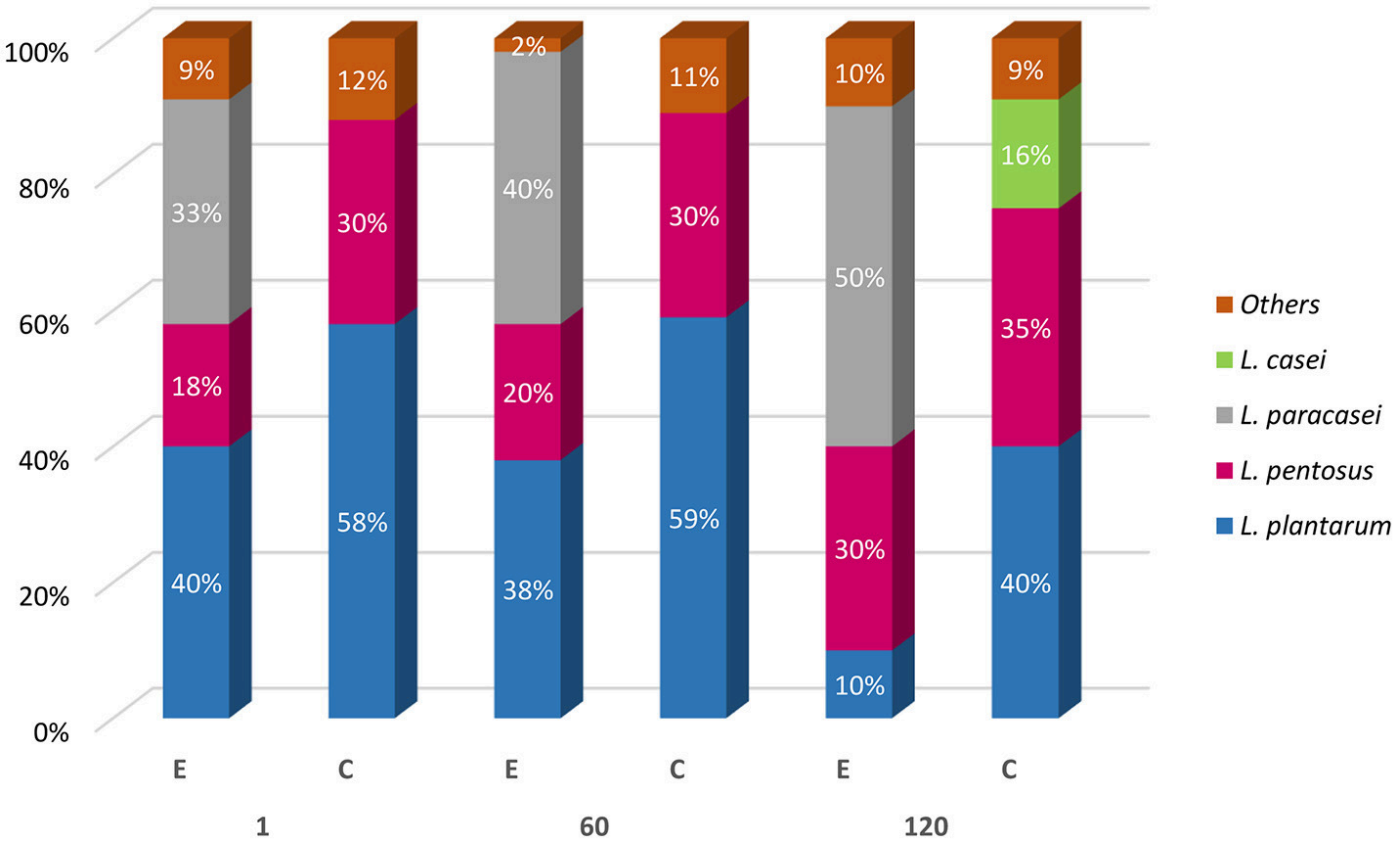
Molecular identification of *Lactobacillus* spp. isolates from experimental (E) and control (C) samples at 1, 60, and 120 days of fermentation.

Four hundred isolates from MRS plates were considered lactobacilli based on their positive Gram reaction, non-motility, absence of catalase activity, and spore formation, and rod or coccal shape. Presumptive lactobacilli were identified by using multiplex PCR and were ascribed to *L. plantarum, L. pentosus, L. paracasei*, and *Lactobacillus casei* species, and their occurrence percentage in E and C samples, at 1, 60, and 120 days of fermentation is illustrated in Figure 6. In detail, C samples, as expected, exhibited at both day 1 and after 60 days, a high occurrence (70%) of *L. plantarum* accompanied by *L. pentosus* (30%). After 120 d, a slight occurrence (20%) of *L. casei* was detected. In E samples, 40% of isolates were ascribed to both *L. paracasei* and *L. plantarum*. The remaining 20% was identified as *L. pentosus*. Similar occurrence was revealed after 60 d of fermentation. Different species occurrence was highlighted at 120 days, since the majority of the isolates were identified as *L. paracasei* (60%), followed by *L. pentosus* (30%) and *L. plantarum* (10%) (Figure 6). It is interesting to note that among the 60 isolates identified as *L. paracasei*, the majority of the occurrence was revealed in E sample at 5% of salt (E2).

### Viability of *L. paracasei* N24 strain through table olives fermentation

**Figure 7:**
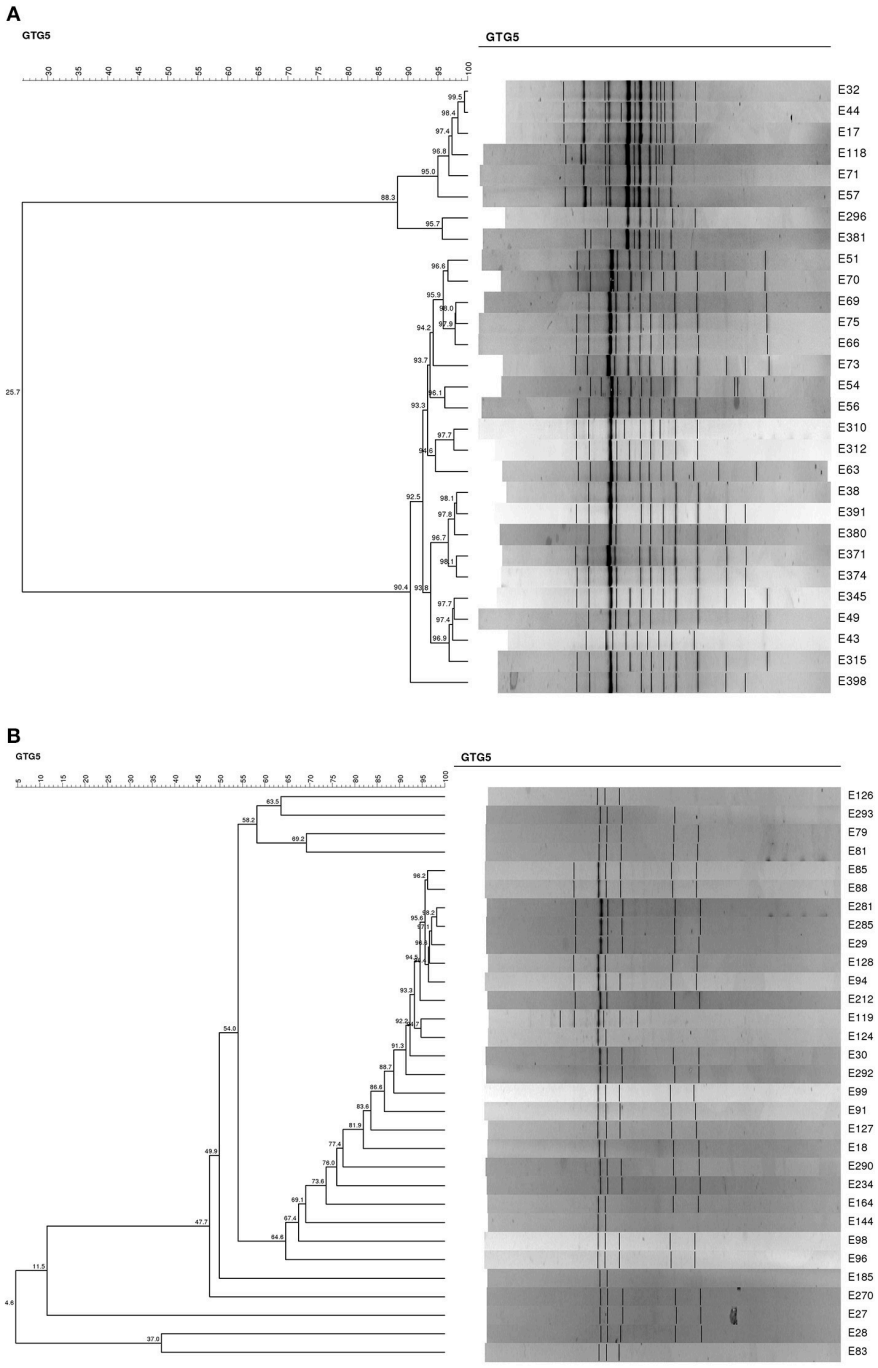
**(A)** Dendrogram generated after cluster analysis of the digitized (GTG)_5_-PCR fingerprints of the lactobacilli isolated from experimental table olives at 60 and 120 days. **(B)**. Dendrogram generated after cluster analysis of the digitized (GTG)_5_-PCR fingerprints of the lactobacilli isolated from experimental table olives at 60 and 120 days.

In order to evaluate the viability of *L. paracasei* strain N24 in the experimental table olives at 60 and 120 d, DNA of 60 strains was submitted to REP-PCR. Results are reported in Figures 7A,B. All strains clustered together with the *L. paracasei* N24 strain (with a percentage of similarity higher than 85%), with the exception of 17 strains, which exhibited different profiles.

### Sensory data

Results of sensory analysis are reported in Table 3. No off-odors were detected in any samples as inferred by the low scores of the taste panel for this organoleptic perception. Overall, regarding the gustatory sensations (acidity, saltiness, and bitterness), differences among E and C samples at different salt content were detected, with the exception of bitterness descriptor. In detail, E samples received similar and moderate values in acidity, while C ones exhibited a higher score, with the highest value for the sample with 8% of salt (C4). A similar trend was observed for the saltiness score, with lower value in E samples. Only samples made adding 8% of salt (E4 and C4) showed significant differences, registering a saltiness score of about 8.1. Regarding kinaesthetic sensations (hardness, fibrousness, and crunchiness) no statistical differences were achieved among samples, registering a mean score of 7.2. Finally, E olives received higher scores for the overall acceptability descriptor, with the exception of E1 sample, which showed an average score similar to those obtained by C samples. Experimental E2 sample exhibited the highest overall acceptability score (8.8 ± 0.82).

**Table 3:** Sensory data of the experimental (E) and control (C) table olives.

**Samples**	**Descriptors**													
	**Abnormal fermentation**							**Gustatory sensations**			**Kinaesthetic sensations**			**Overall acceptability**
	**Musty**	**Rancid**	**Cooking Effect**	**Soapy**	**Metallic**	**Earthy**	**Winey-Vinegary**	**Acidity**	**Saltiness**	**Bitterness**	**Hardness**	**Fibrousness**	**Crunchiness**	
E1	1.1 ± 0.29^a^	0.4 ± 0.28^a^	0.7 ± 0.16^a^	0.2 ± 0.12^ab^	0.5 ± 0.12^a^	1.3 ± 0.12^a^	0.9 ± 0.31^a^	3.7 ± 0.36^a^	6.1 ± 0.29^a^	2.6 ± 0.12^a^	5.3 ± 0.61^ab^	3.2 ± 0.12^a^	6.4 ± 0.23^a^	8.5 ± 0.12^a^
E2	1.3 ± 0.49^a^	0.3 ± 0.25^a^	0.5 ± 0.17^a^	0.0 ± 0.05^ab^	0.6 ± 0.12^a^	1.1 ± 0.29^a^	1.1 ± 0.23^a^	3.6 ± 0.3^a^	6.3 ± 0.41^a^	2.7 ± 0.09^a^	6.9 ± 0.05^bc^	3.3 ± 0.14^a^	6.7 ± 0.16^a^	8.8 ± 0.82^a^
E3	1.4 ± 0.24^a^	0.4 ± 0.23^a^	0.7 ± 0.21^a^	0.3 ± 0.16^a^	0.5 ± 0.05^a^	1.3 ± 0.26^a^	1.1 ± 0.28^a^	3.7 ± 0.12^a^	6.5 ± 0.29^ab^	2.8 ± 0.37^a^	5.2 ± 0.78^a^	3.5 ± 0.34^a^	6.3 ± 0.12^a^	8.5 ± 0.21^a^
E4	1.3 ± 0.53^a^	0.5 ± 0.28^a^	0.8 ± 0.12^a^	0.2 ± 0.05^ab^	0.5 ± 0.29^a^	1.1 ± 0.61^a^	0.8 ± 0.41^a^	4.5 ± 0.21^ab^	7.3 ± 0.41^ab^	2.6 ± 0.12^a^	5.3 ± 0.16^a^	3.3 ± 0.21^a^	6.3 ± 0.31^a^	8.2 ± 0.29^a^
C1	1.3 ± 0.12^a^	0.2 ± 0.21^a^	0.7 ± 0.16^a^	0.3 ± 0.05^ab^	0.7 ± 0.21^a^	1.1 ± 0.58^a^	0.9 ± 0.37^a^	5.3 ± 0.61^bc^	6.4 ± 0.05^ab^	5.3 ± 0.08^b^	7.2 ± 0.08^c^	3.4 ± 0.35^a^	6.6 ± 0.43^a^	7.9 ± 0.33^a^
C2	1.4 ± 0.21^a^	0.3 ± 0.34^a^	0.6 ± 0.16^a^	0.4 ± 0.16^b^	0.7 ± 0.12^a^	1.3 ± 0.29^a^	1.1 ± 0.25^a^	5.4 ± 0.86^bc^	6.4 ± 0.90^ab^	5.5 ± 0.86^b^	7.3 ± 0.37^c^	3.5 ± 0.11^a^	6.7 ± 0.38^a^	7.7 ± 0.61^a^
C3	1.4 ± 0.37^a^	0.4 ± 0.24^a^	0.7 ± 0.21^a^	0.2 ± 0.05^ab^	0.8 ± 0.33^a^	1.3 ± 0.54^a^	1.2 ± 0.53^a^	5.4 ± 0.37^bc^	6.3 ± 0.37^a^	5.3 ± 0.78^b^	7.2 ± 0.57^c^	3.8 ± 0.23^a^	6.5 ± 0.29^a^	7.6 ± 0.57^a^
C4	1.3 ± 0.12^a^	0.4 ± 0.40^a^	0.7 ± 0.21^a^	0.2 ± 0.12^ab^	0.5 ± 0.37^a^	1.2 ± 0.61^a^	1.2 ± 0.61^a^	6.2 ± 0.53^c^	7.7 ± 0.12^b^	5.2 ± 0.53^b^	7.2 ± 0.53^c^	3.2 ± 0.40^a^	6.5 ± 0.26^a^	7.6 ± 0.57^a^

*a–c for each descriptor, in the same column, with different superscript letters are significantly different (P < 0.05)*.

## Discussion

Consumer's acceptance and attitude toward functional foods determine the market success, which is growing steadily, mainly toward vegetables, fruits, and cereal products due to vegetarianism emergence, lactose intolerance, cholesterolemia, and food allergies (Granato et al., 2010; Ranadheera et al., 2010; Peres et al., 2012; Martins et al., 2013). Among the non-dairy functional foods, table olives represent a good food matrix to carry active viable bacteria into the gastrointestinal tract (Lavermicocca et al., 2005; Valerio et al., 2006; Abriouel et al., 2012; Randazzo et al., 2017). Table olives are considered functional food because of their nutritional value related to the presence of phenolic compounds and monounsaturated fatty acids (Buckland and Gonzalez, 2015). Nevertheless, their preparation relies on the use of NaCl as the main ingredient of the brine especially for reducing undesirable spoilage and pathogenic microorganisms ensuring, thus, the microbiological safety and quality of the final product (Taormina, 2010; Albarracín et al., 2011). In recent years, public health and regulatory authorities have recommended the reduction of dietary intake of sodium because of its association to hypertension [World Health Organisation (WHO), 2003, 2007], and to cardiovascular diseases (Ortega et al., 2011). In Mediterranean regions, populations eat considerably high amounts of table olives and, subsequently, ingest greater amounts of salt; hence, NaCl reduction in table olives is strongly recommended. Recently, several studies have been focused on the replacement of NaCl with other salts, such as KCl and CaCl_2_ (Bautista-Gallego et al., 2010, 2013a,b; Mateus et al., 2016; Zinno et al., 2017), and results are not fully in areement. In particular, while Bautista-Gallego et al. (2010) showed that *Enterobacteriaceae* growth was slightly stimulated by high CaCl_2_ contents, Mateus et al. (2016) revealed that the presence of potassium and calcium chlorides in the brines caused an increase of the enterobacteria death rate. Up to now only De Bellis et al. (2010) have proposed to study table olive processing at reduced NaCl concentration, without any salt replacement. Based on our previous reported data (Randazzo et al., 2017), with the aim to set up functional table olives from Nocellara Etnea cultivar, in the present study the fermentation was carried out at different salt contents (4, 5, 6, and 8%), by using starter cultures constituting of the promising probiotic strain *L. paracasei* N24 and by the strain *L. plantarum* UT2.1. The fermentation without the use of starter cultures was used as control. It is well established that both salt content and pH value are the main parameters controlling the pathogens growth in fermented products, such as *C. botulinum*. Taormina (2010) has already reported that the probability of growth and toxin production of *C. botulinum* at 5% NaCl decreased as the pH and storage temperature was decreased. In this context, our data reveled that all experimental brines have had a pH value below 4.5, with the exception of control sample C4, and a constant temperature of ca 18°C, which guarantee the pathogens growth inhibition. Overall, in contrast to De Bellis et al. (2010), who observed alterative processes in spontaneous fermentation at 4% of NaCl, in the present study, samples treated with different salt content obtained similar scores in terms of “abnormal fermentation” sensory attribute. Slight sensory differences were detected only for bitterness and acidity descriptors among experimental and control samples, with a higher score in control ones. These differences could be therefore attributed to the added starter cultures. In particular, the higher contents of acetic acid, ethanol, and phenols, associated to vinegary, fatty smell, and bitter aroma, respectively, in control samples, could justify the highest acidity and bitterness scores registered by panelists. On the contrary, it is interesting to note that the experimental sample at 5% of NaCl (E2) showed the highest overall acceptability score and, based on the similarity tree, this sample exhibited a unique profile. Even in the experimental samples a high amount of propionic acid was detected, panelists did not reveal any off-flavors, indicating that the defect generated by propionic acid was probably hidden by other compounds such as esters, as indicated by Blana et al. (2014). In fact, among total VOCs, esters showed an occurrence percentage higher in experimental samples rather than in control samples, contributing to more pleasant flavors (Sabatini et al., 2008). Evaluating both PCA data on VOCs and similarity tree, brine samples were mainly grouped based on fermentation time, in discordance to Blana et al. (2014), that demonstrated the importance of salt content on the fermentation profiles. The present study revealed that salt contents slightly influences the metabolome of table olives; however, the overall characteristics of the final products were strongly time-dependent. In addition, the salt content did not affect the performances of *L. paracasei* N24 and *L. plantarum* UT2.1 starter cultures used. They were effective in accelerating the fermentation process, quickly reducing the pH from the 7th day of fermentation, inhibiting spoilage bacteria in all experimental samples. In fact, at the end of the fermentation, *Enterobacteriaceae* were countable only in control samples. This microbial group, as suggested by Medina-Pradas et al. (2017), can negatively influence the quality and safety of table olives, causing gas pockets spoilage, or producing metabolites that affect the final aroma. Moreover, the present work allowed to asses that starters, rather than NaCl replacement with CaCl_2_ and/or KCl, as discussed by Mateus et al. (2016), has an effect on the reduction of the yeast population. In fact, in the present study yeast decreasing could be attributed to the intense competition between LAB and yeasts for nutrients. It is noteworthy that yeasts are involved in the VOCs formation; nevertheless, a high occurrence of this microbial group could be responsible of undesirable fermentation. In the present study, yeasts were positively correlated with the main alcohols and phenols detected, which could generate off-flavors. Hence, the use of starter cultures is strongly recommended in table olives fermentation also in order to inhibit spoilage bacteria and control the autochthonous yeast growth. Evaluating the lactobacilli behavior, *L. plantarum* and *L. pentosus* were the main species detected at the end of the fermentation in all samples, confirming their key role in table olive fermentation. In addition, a high survival rate of the promising probiotic N24 strain was depicted in all experimental samples. This evidence confirms its technological suitability to be used as starter in olive fermentations as well as its ability to survive during the process regardless of brine salt concentration. In addition, the promising probiotic *L. paracasei* N24 strain exhibited the highest occurrence in experimental sample at 5% of salt (E2). The latter was clearly separated from the remaining treatments, exhibiting a unique metabolomics profile, which generate sensorial traits appreciate by panelists. Hence, data of the present investigation revealed promising perspectives for the application of *L. paracasei* N24 strain as starter cultures for the production of table olives with increased added value.

## Conclusion

Results of the present study demonstrated that both brine microbial population and VOCs were slightly affected by salt content while a strong influence was determined by time of fermentation. The reduction of NaCl content, without any replacement with other salts resulted in a successful fermentation of Nocellara Etnea table olives. The final products fulfilled microbiological criteria and exhibited more appreciate sensorial characteristics. In addition, the formulation of probiotic table olives with low salt content is healthier and more suitable for consumers at risk of hypertension, opening new perspectives for their production at industrial scale.

## Author contributions

AP, AT, and KV performed the experiments, and analyzed data. AP and CR wrote the manuscript. MD, CR, and CC designed the study and contributed to data interpretation. AT and KV co-wrote the manuscript.

### Conflict of interest statement

The authors declare that the research was conducted in the absence of any commercial or financial relationships that could be construed as a potential conflict of interest.

## Supplementary material

Figure S1 Total phenolic content (mg/l) of control (C) experimental (E) brine samples at 60 and 120 days of fermentation.
