# Supplementary figures and images for: Fermentation of Nocellara Etnea Table Olives by Functional Starter Cultures at Different Low Salt Concentrations
Source: Front Microbiol. 2018 Jun 5;9:1125. doi: 10.3389/fmicb.2018.01125 (PMC5996112; doi:10.3389/fmicb.2018.01125)

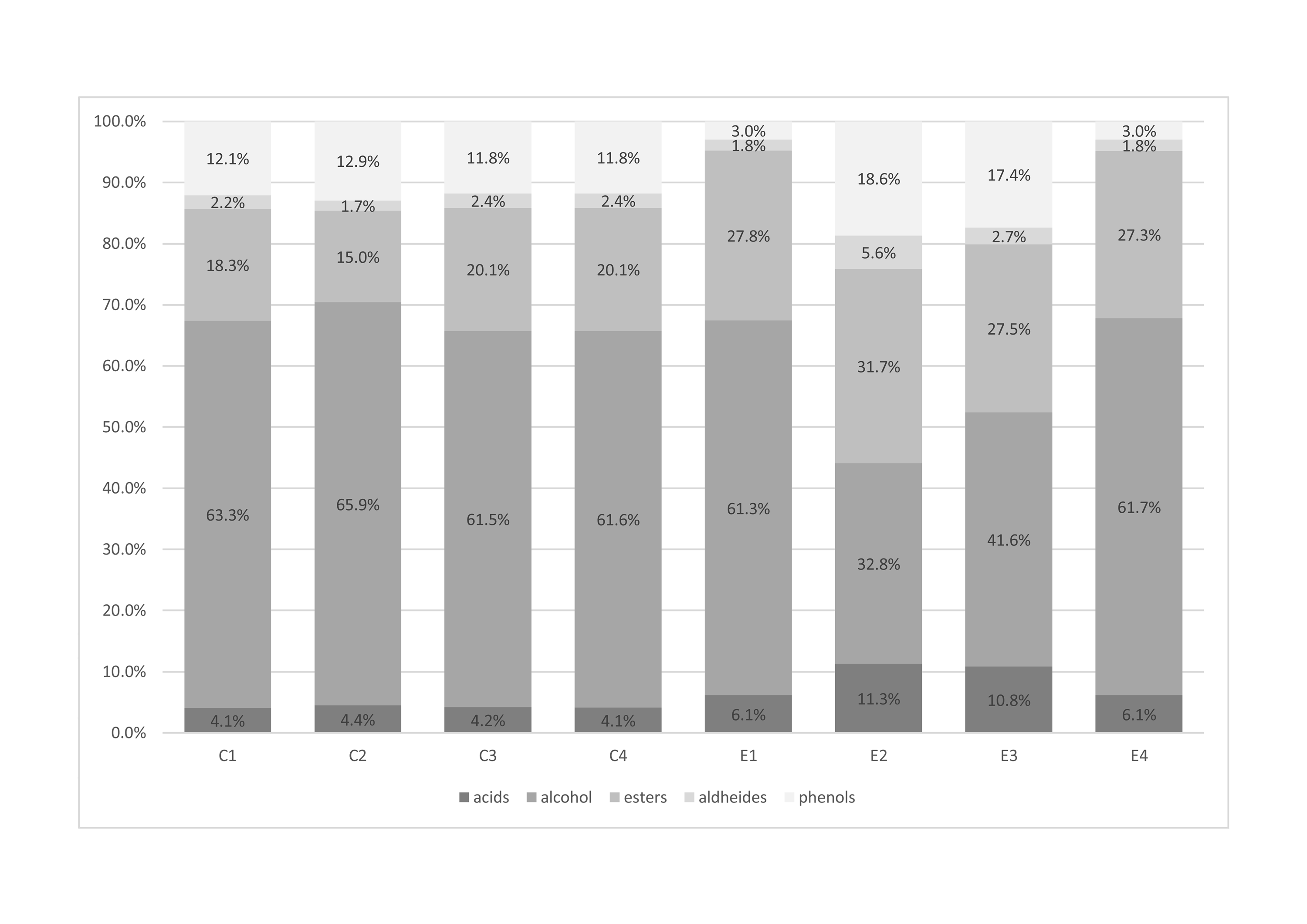

Supplement: Figure S1 — Total phenolic content (mg/l) of control (C) experimental (E) brine samples at 60 and 120 days of fermentation. [file Image_1.TIF]
